# Effect of Baseline Impedance in Radiofrequency Delivery on Lesion Characteristics and the Relationship Between Impedance and Steam Pops

**DOI:** 10.3389/fcvm.2022.872961

**Published:** 2022-04-27

**Authors:** Lijuan Qu, Min Guo, Meng Sun, Rui Wang, Nan Zhang, Xin Li

**Affiliations:** ^1^Department of Cardiovascular Medicine, Shanxi Bethune Hospital, Third Clinical Medical College of Shanxi Medical University, Taiyuan, China; ^2^Department of Cardiology, First Hospital of Shanxi Medical University, Taiyuan, China

**Keywords:** radiofrequency ablation, lesion characteristics, impedance, steam pop, cardiovascular medicine

## Abstract

**Objective:**

To explore the effects of baseline impedance (R) and power (P) on radiofrequency ablation (RFA) lesion characteristics and their correlation with steam pops using ThermoCool SmartTouch-SF (STSF) catheters in the porcine heart.

**Method:**

A porcine left ventricle was submerged in 37°C saline *ex vivo*, and the experiment was performed with various *P* (*P* = 30, 40, 50, and 60 W) and multiple *R* loads (*R* = 80–100, 100–140, 140–180, and 180–220 Ω) to reach the target ablation index (AI; AI = 350, 450, and 500) or reach the target ablation time using a fixed contact force (CF; CF = 10–15 g) and the same saline irrigation (30 W/8 ml/min or 40–60 W/15 ml/min), repeated five times under each condition.

**Results:**

The surface diameter, maximum diameter, depth, and volume of the lesions were strongly correlated with the AI (*P* = 40 W, *R* = 100–140 Ω, CF = 10–15 g) (*r* = 0.5412; *r* = 0.7889; *r* = 0.9366; and *r* = 0.913, respectively; all *p* < 0.05). As the value of *R* increased, the maximum diameter, depth, and volume of the lesions significantly increased (AI = 350, *P* = 30 W). Moreover, the higher the baseline value of *R*, the greater the absolute value of the *R* decrease (*r* = 0.9035, *p* < 0.05, *Y* = 0.2759 × *X* – 18.33). Under high power and high impedance, the occurrence rate of steam pops was high (*P* = 60 W, *R* = 180–220 Ω, AI when a steam pop occurred: 480 ± 26.5, ablation time: 11.29 ± 1.04 s).

**Conclusion:**

Radiofrequency catheter ablation (RFCA) in power-controlled mode resulted in various lesion characteristics that were related to diverse baseline Rs. In addition, the incidence of steam pops was strongly correlated with high baseline *R* and high *P*.

## Introduction

Increasing attention is being focused on improving the efficacy and safety of radiofrequency catheter ablation (RFCA) in contemporary clinical work, and RFCA is becoming the preferred choice to treat various tachycardias. The ThermoCool STSF catheter in RFCA is often used in power-controlled mode (1).

Previous studies have confirmed that power (P), contact force (CF), and time are all vital factors in effectively ablating a lesion (2–6). The above relative factors are essential and are investigated as contributors to the ablation index (AI); this is a novel ablation guidance parameter combining these factors in a weighted formula that has been reported to predict the characteristics of a lesion (4, 7). However, the AI did not take into account the effects of baseline impedance, impedance drop, and the type of perfusate on the ablation lesion. In addition, the patient was connected in a series in the RFCA circuit to ensure that the circuit was complete. The relationship between the *P* and the current is affected by the impedance (*R*); the *P* is equal to the square of the current (*I*^2^) multiplied by the *R*: *P* = *I*^2^ × *R*. The characteristics of the lesion may be influenced by diverse Rs because of the various energy deliveries during RFCA, even if they are in the same power-controlled setting. There are several Rs in the circuit: the patient's *R* (one part exists in the tissue and the blood flow surrounding the ablation catheter, and another part exists between the catheter tip and the patch placed on the skin of the buttocks), the catheter *R*, and the generator *R* in the circuit. Even different conditions of the cell lead to different myocardial Rs (1, 8, 9). In this study, the influence of the *R* on the RFCA lesion characteristics in a power-controlled setting and the relationship between the *R* and steam pops will be investigated.

## Methods

### *Ex vivo* Model

**Figure 1:**
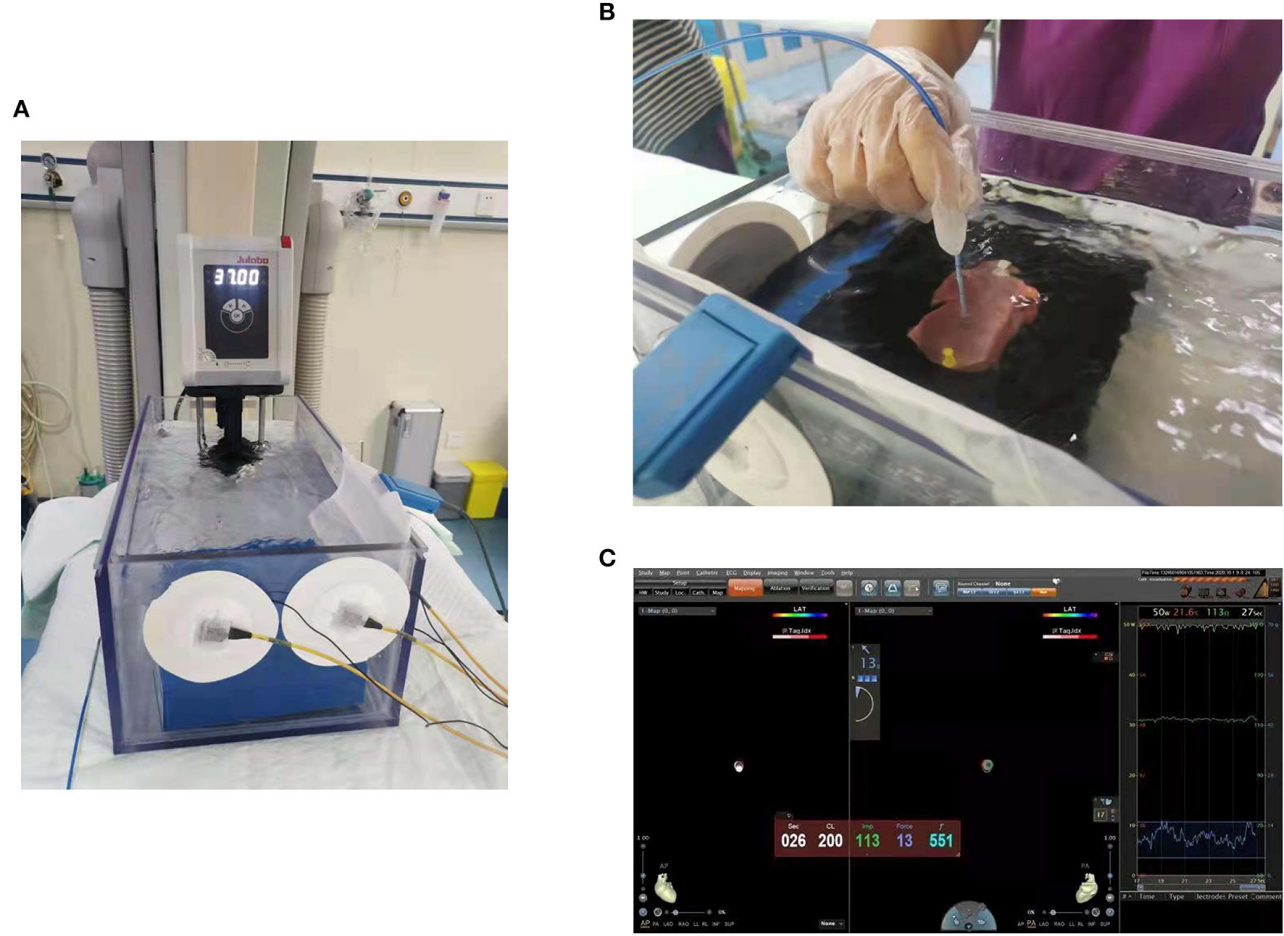
An *ex vivo* experimental model. **(A)** This model consisted of saline with a circulating pump with the temperature maintained at 37°C. **(B)** The approach that manually fixed the catheter so that it met the tissue to produce the corresponding catheter force. **(C)** The catheter stability using the CARTO3 system.

A total of 30 fresh pig hearts, with an average weight of 0.59 ± 0.14 kg, were purchased from Taiyuan slaughterhouse, Shanxi Province, China. Figure 1A shows the *ex vivo* experimental model. Freshly purchased porcine heart ventricles were incised and fixed on a rubber platform in a transparent container filled with saline with a circulating pump (JULABOMB Heating Immersion Circulator, JULABO GmbH, Seelbach, Germany) containing solution mixed from 0.9% NaCl solution and purified water, maintaining a temperature of 37°C. The baseline *R* was adjusted by adding crystalline salts or purified water. The ablation system included the SmartAblate radiofrequency generator and the Carto3 mapping system (Biosense Webster). A ThermoCool STSF ablation catheter (Biosense Webster, Diamond Bar, CA) was manually applied perpendicular to the epicardium (Figure 1B). The relative information of catheter, such as the stability, ablation curve, and the baseline *R* was displayed on the monitor using the CARTO3 system (Figure 1C).

### Ablation Strategy

The experiment was performed with multiple *R* loads (low impedance = 80–100, medium impedance = 100–140, high impedance =140–180, and very high impedance = 180–220 Ω), with various Ps (30, 40, 50, and 60 W), and a fixed CF at 10–15 g, reaching the target AIs of 350, 450, and 500. RFCA used the same saline irrigation at 8 ml/min, while *P* was 30 W or 15 ml/min in other overpass 30 W *P* settings. The ablation at each setting was repeated five times unless an acoustic steam pop occurred. In cases where a steam pop occurred, RFCA was conducted five additional times and the relevant data were recorded. If the observation end point (reaching the target AI or if steam pops occurred) did not occur after 120 s, the RFCA was stopped. In addition, ablation at fixed power (30, 40 W), fixed force (15–20 g), and 1 min duration for 5–10 ablations were conducted in every set. The correlation between the lesion characteristics and the ablation parameters was explored.

### Lesion Measurements

**Figure 2:**
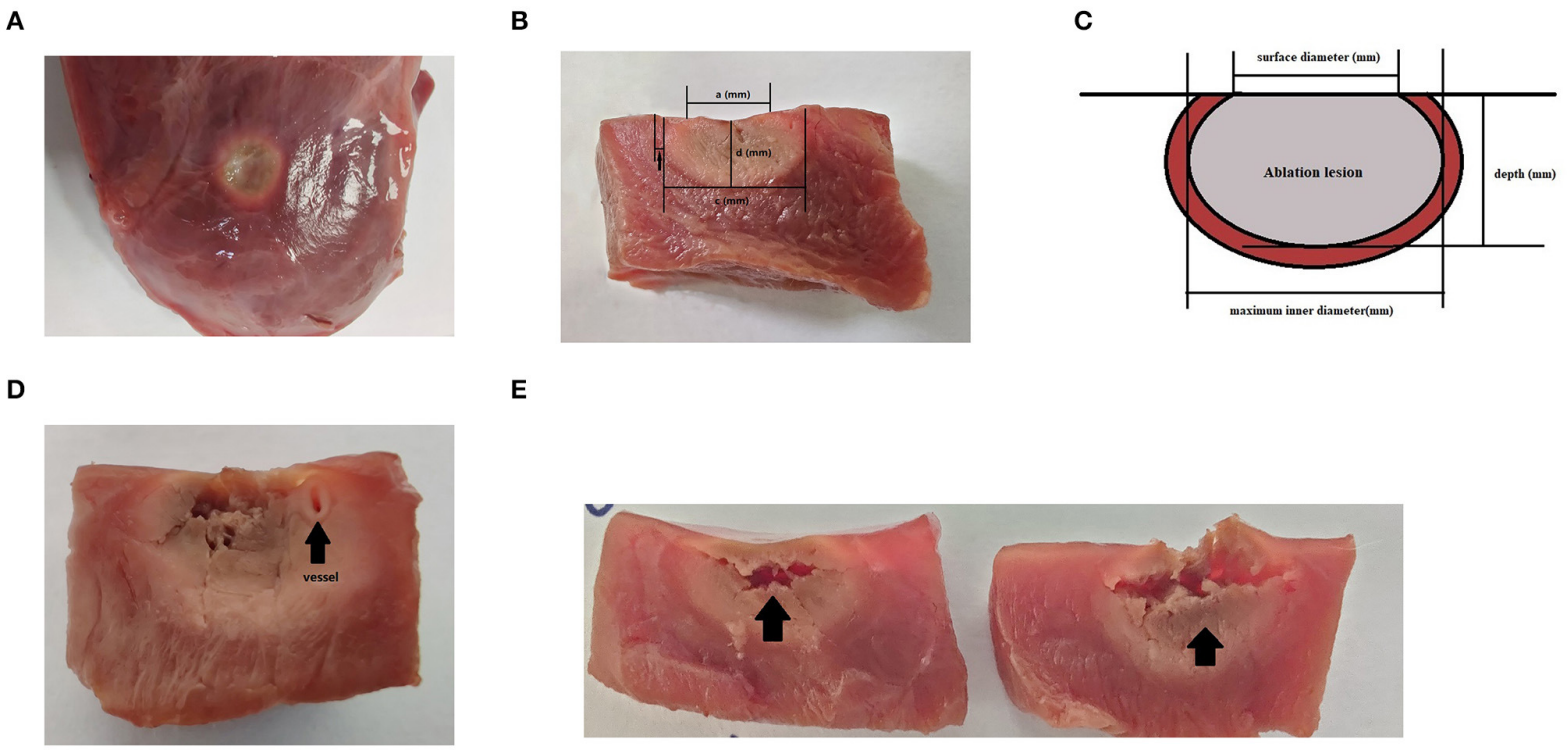
Lesions after 20 min of waiting after radiofrequency catheter ablation (RFCA). **(A)** The surface view of the ablation lesion, which is a perfect circle. The pale part is the ablation lesion, while the area indicated by black arrows is the hyperemia and edema area. **(B)** The transverse section of an oval ablation lesion. The lesion border was defined as the tissue color. **(C)** A schematic diagram of the measurement diameter of the ablation lesion, where “a” represents the surface diameter, “c” represents the maximum diameter, and “d” stands for depth. Each diameter line was measured using an electronic vernier caliper. **(D)** A steam pop with a small blood vessel visible at the arrowhead. **(E)** A steam pop with a horizontal tear in the tissue.

After the ablation, the tissue was exposed to air for 20 min to allow the hyperemia and edema zone to become clear. Then, the surface diameter (*a*), depth (*d*), and maximum inner diameter (*c*) of each lesion were measured using an electronic vernier caliper. Each diameter was measured three times. The lesion volume was calculated (10) as follows: volume = [1.33 × π × *d* × (*a*/2) × (*c*/2)]/2. All the lesion measurements were collected independently by an investigator who was blinded to the ablation parameters (Figure 2).

### Statistical Analysis

The data were analyzed with SPSS™ Statistics v23.0 statistical software. Descriptive statistics were reported as the means ± standard deviations (SDs) for the continuous variables. Comparisons between the groups for the normally distributed variables were made by independent samples one-way analysis of variance (ANOVA) *t*-tests. Spearman's correlation coefficients were used to determine the correlations in the data. The value of *p* < 0.05 was considered to be statistically significant.

## Results

### General Biophysical Analysis

**Figure 3:**
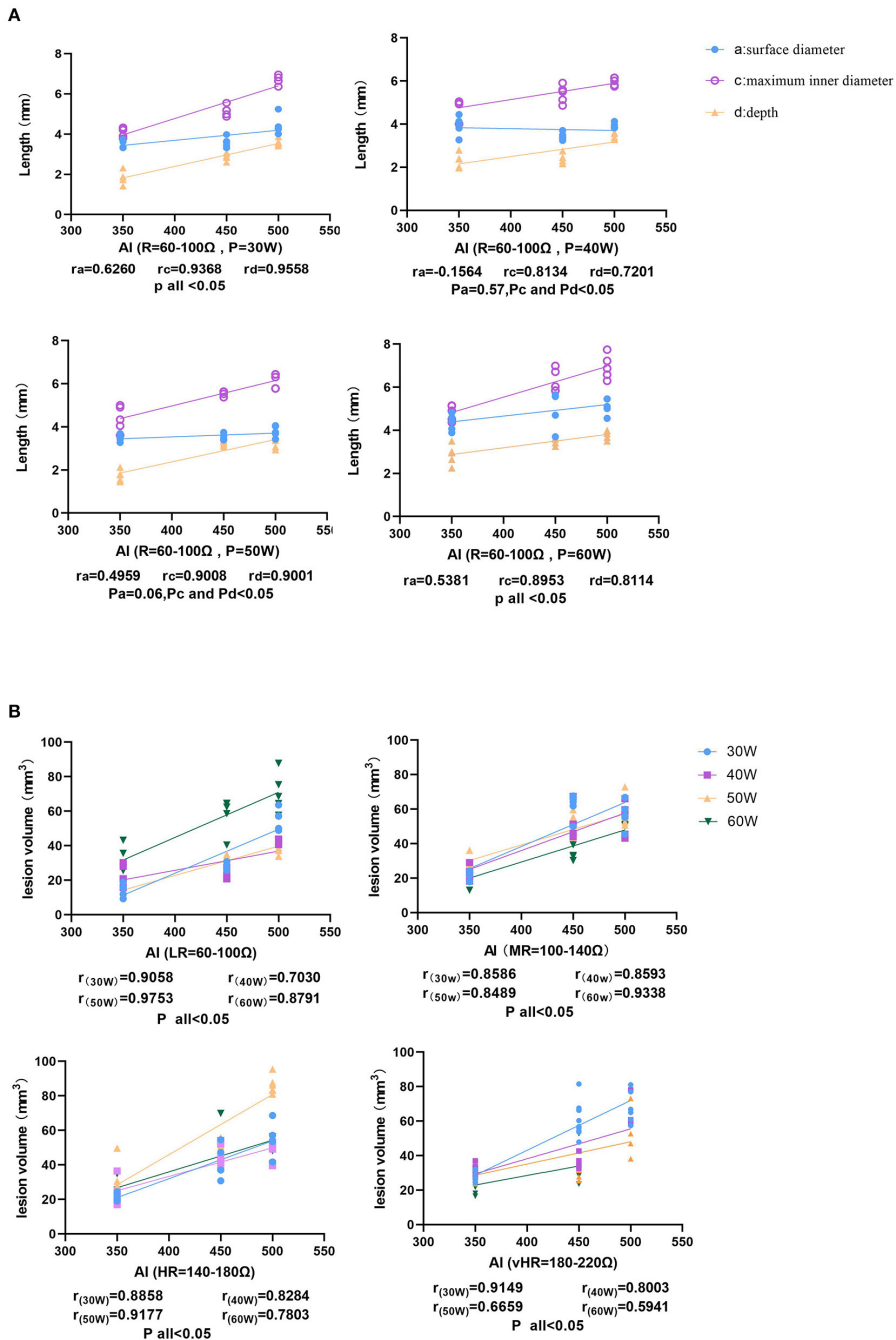
The correlation between ablation index (AI) and lesion characteristics. **(A)** The size of the ablation lesions at the same baseline *R* (60–100 Ω) and different *P* settings (30, 40, 50, and 60 W), showing that the surface diameter of the ablation lesions increases with AI, the maximum inner diameter, and the depth, showing an increasing trend. **(B)** The relationship between volume and AI at different baseline impedances.

In total, 418 lesions, including 149 lesions with steam pops and 8 lesion ablations that were stopped after 120 s, were analyzed. Obvious and uniform hyperemia and edema zones could be seen around the ablation injury (Figure 2), whereas the edema zones around the steam pops were uneven or fractured. According to the statistical analysis, the surface diameter, maximum diameter, depth, and volume of the lesions were strongly correlated with the AI. In different basic impedance groups, the volume of ablation injury increased gradually with the increase of target AI (*P* = 40 W, baseline *R* = 100–140 Ω, and CF = 10–15 g) (*r* = 0.5412; *r* = 0.7889; *r* = 0.9366; and *r* = 0.913, respectively; all *p* < 0.05) (Figure 3).

### Correlation Between the Baseline *R* and Lesion Characteristics

**Figure 4:**
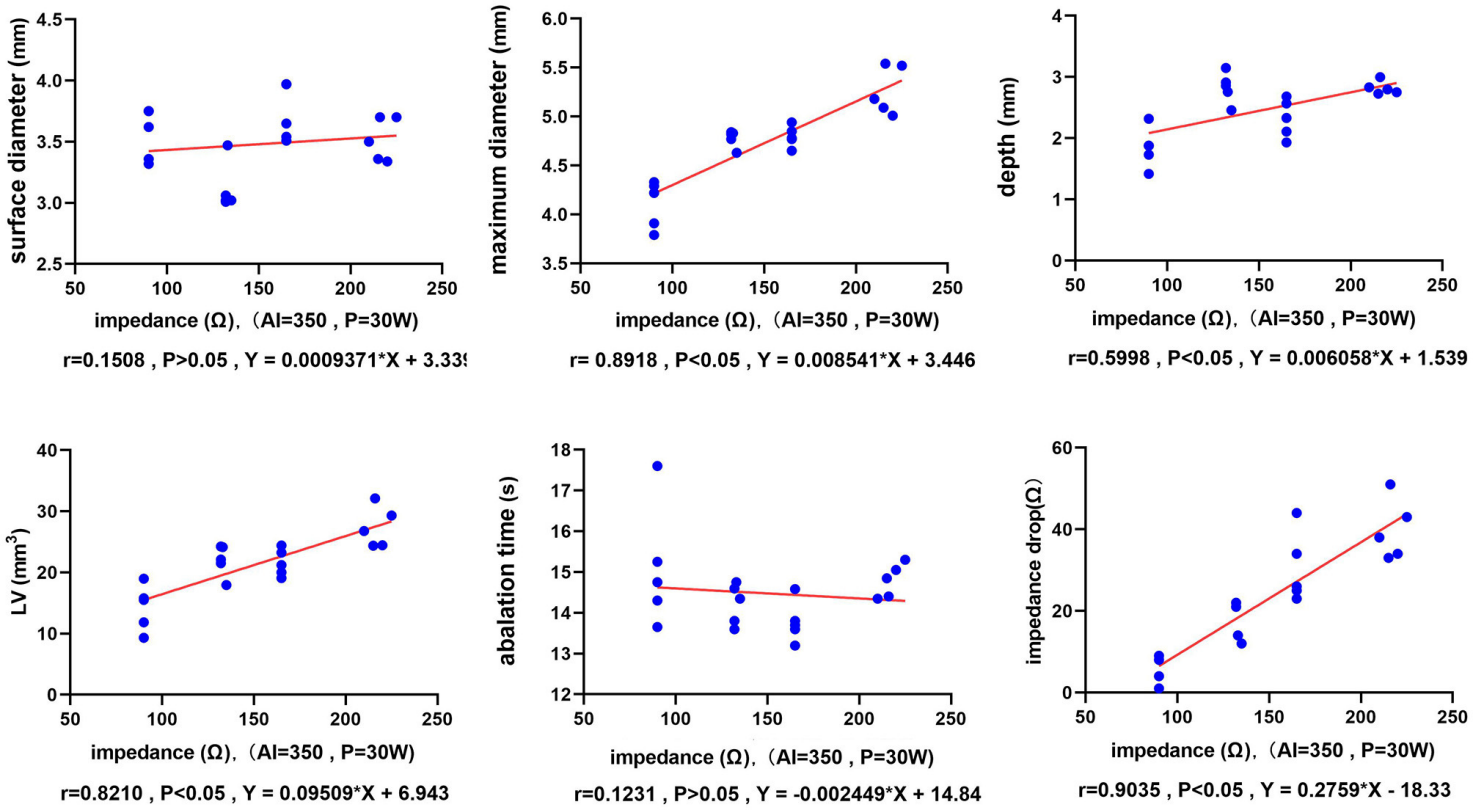
The relationship between the ablation parameters and the lesion dimensions. The six-line graphs show the correlations between the baseline *R* and the ablation time (T), the *R* drop, and the lesion size. Each dot represents an individual radiofrequency-ablated lesion. The red line is the fitted linear regression line.

**Figure 5:**
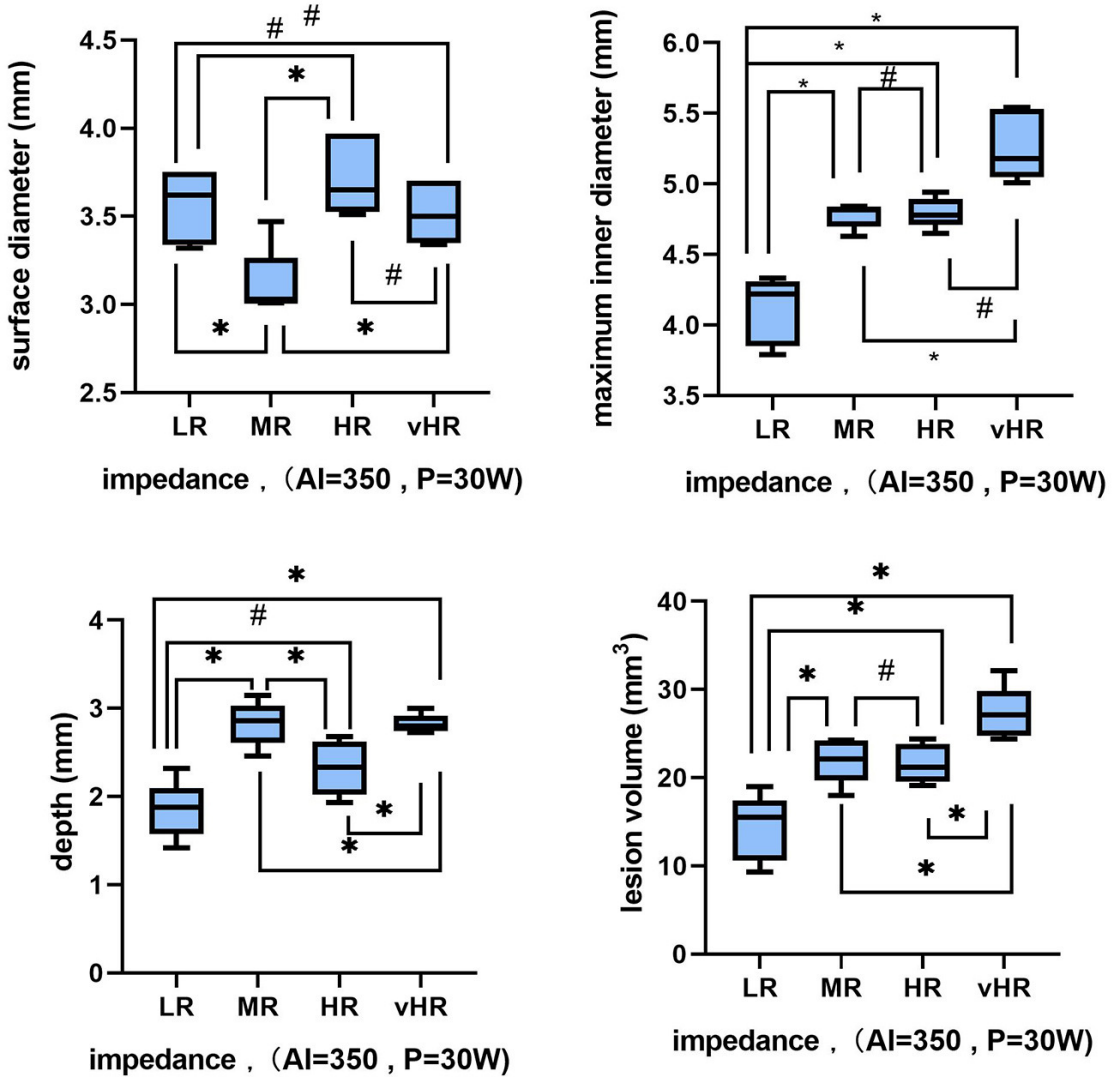
Variations in the lesion characteristics during baseline *R* changes (low impedance = 80–100 Ω, medium impedance = 100–140 Ω, high impedance =140–180 Ω, and very high impedance = 180–220 Ω) when *P* = 30 W and AI = 350. Each box represents the interquartile range (IQR) of each measure, with the middle horizontal line representing the median. The vertical lines denote the minimum and maximum values. The symbols denote whether these changes are statistically significant: “*” means *p* < 0.05 and “#” means *p* > 0.05.

Figure 4 shows that as the value of *R* increased, the lesion maximum diameter, depth, and volume significantly increased at a fixed AI target = 350 and a *P* of 30 W [*r*_(maximum diameter vs. impedance)_ = 0.8918, *p* < 0.05, *Y* = 0.008541 × *X* + 3.446; *r*_(depth vs. impedance)_ = 0.5998, *p* < 0.05, *Y* = 0.006058 × *X* + 1.539; and *r*_(volume vs. impedance)_ = 0.8210, *p* < 0.05, *Y* = 0.09509 × *X* + 6.943]. In addition, there were no statistically significant differences in the surface diameter or ablation time to reach the target AI [*r*_(surface diameter vs. impedance)_ = 0.1508, *p* = 0.52; and *r*_(ablation time vs. impedance)_ = −0.1232, *p* = 0.60]. However, the *R* drop was strongly correlated with the baseline *R* (*r* = 0.9035, *p* < 0.05, *Y* = 0.2759 × *X* – 18.33). The lesion information of each group is shown in Table 1 and Figure 5 (same target AI of 350).

**Table 1:** Lesion information of every group (AI = 350), which is represented by the means ± SDs.

	***a*** **(mm)**	***c*** **(mm)**	***d*** **(mm)**	**LV (mm^**3**^)**
**AI = 350/*P* = 30 W**				
60–100	3.56 ± 0.20	4.10 ± 0.24	1.84 ± 0.32	14.29 ± 3.75
100–140	3.11 ± 0.19	4.77 ± 0.87	2.82 ± 0.25	21.99 ± 5.58
140–180	3.72 ± 0.22	4.79 ± 0.10	2.32 ± 0.31	21.59 ± 0.21
180–220	3.52 ± 0.17	5.26 ± 0.24	2.82 ± 0.10	27.40 ± 0.31
**AI = 350/*P* = 40 W**				
60–100	3.93 ± 0.43	4.80 ± 0.44	2.31 ± 0.33	23.03 ± 2.93
100–140	3.72 ± 0.19	4.98 ± 0.46	2.40 ± 0.15	23.39 ± 3.82
140–180	3.65 ± 0.11	5.85 ± 0.24	2.42 ± 0.44	23.47 ± 7.78
180–220	3.66 ± 0.20	5.26 ± 0.24	2.74 ± 0.13	30.85 ± 3.88
**AI = 350/*P* = 50 W**
60–100	3.48 ± 0.15	4.38 ± 0.58	1.71 ± 0.25	13.69 ± 1.27
100–140	4.12 ± 0.23	5.36 ± 0.58	2.54 ± 0.19	29.35 ± 7.1
140–180	3.46 ± 0.60	5.38 ± 0.44	2.80 ± 0.09	27.25 ± 2.7
180–220	3.74 ± 0.28	5.30 ± 0.17	2.84 ± 0.16	29.64 ± 4.44

These results suggest that a larger lesion could be obtained under a medium *P* and a high baseline *R* setting (*P* = 40 W, AI = 350, baseline *R* = 180–220 Ω, maximum volume = 30.85 ± 3.88 mm^3^). However, a higher AI value could not be obtained under a high *P* and high baseline *R* setting because of the steam pops.

### Steam Pop Formation

**Figure 6:**
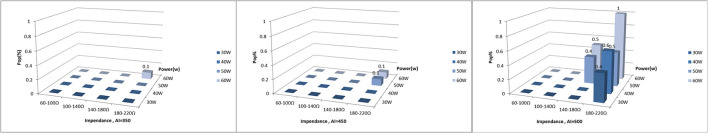
The three bar charts show the proportion of steam pops under different combinations of basic impedance and power when the target AI is 350, 450, and 500, respectively. The *x*-axis represents *R* grouping, the *y*-axis represents the *P* setting, and the *z*-axis represents the proportion of steam pops.

Figure 6 shows the relationship between the incidence of steam pops, the *P*, and the AI. When the target AI was 350, one steam pop occurred at *P* = 60 W and baseline *R* = 180–200 Ω (CF = 12 g, pre-ablation *R* = 217 Ω, final AI = 360, and ablation time = 5.80 s). When the target AI continued to increase, a steam pop appeared in the group with higher *P* and higher *R*, and the incidence of steam pops increased as the baseline *R* increased. In addition, when *P* = 60 W and *R* = 180–220 Ω, a steam pop occurred before AI = 500 (AI: 480 ± 26.5, ablation time: 11.29 ± 1.04 s).

**Figure 7:**
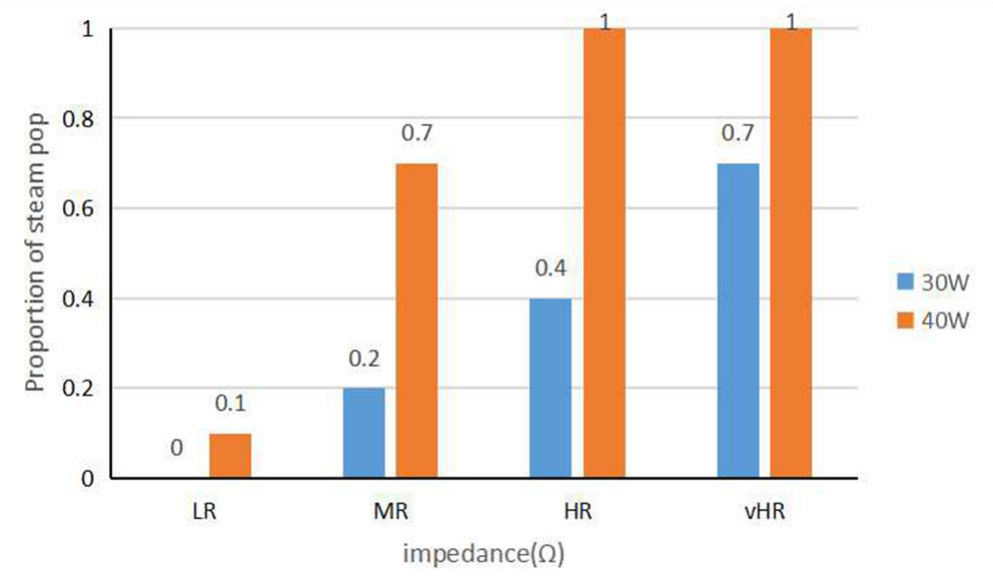
The graph shows the relationship between baseline impedance and the incidence of steam pops at a fixed ablation time.

Figure 7 shows the relationship between baseline impedance and the proportion of steam pop for a fixed time ablation. When the power was 50 or 60 W, the ablation could not last for 60 s because of a premature steam pop. At a fixed ablation time of 60 s, the incidence of steam pops increased with the increasing baseline impedance. When *P* = 30 W, there were no steam pops in the LR group, and the proportion of steam pops in the vHR group was 7/10. When *P* = 40 W, a steam pop occurred in all ablation points in the HR and vHR groups.

**Figure 8:**
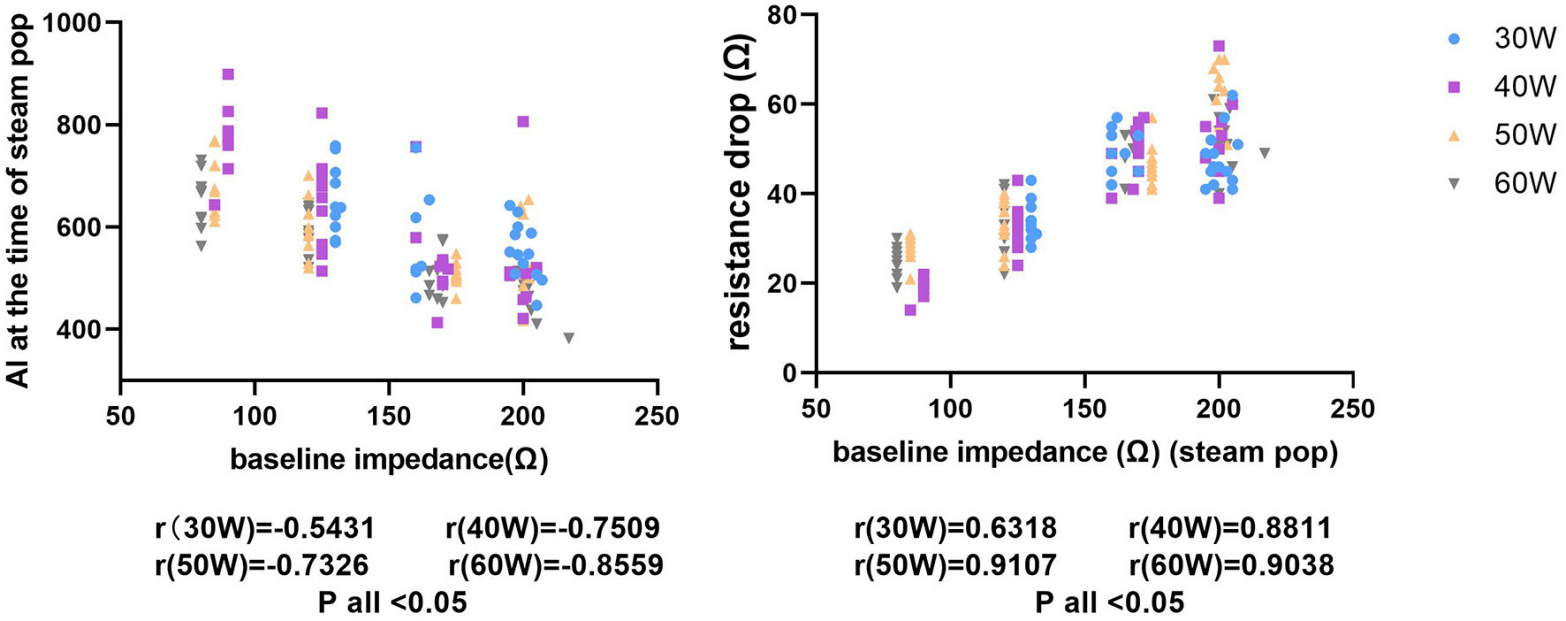
The figure on the left shows the relationship between different baseline impedances and AIs during steam pops. The *x*-axis represents the baseline impedance and the *y*-axis represents the immediate AI when a steam pop occurs. The figure on the right shows the relationship between the baseline impedance and the impedance drop with a steam pop.

Figure 8 shows the correlation between the *R* drop, the AI, and the baseline *R* under a fixed CF (CF = 10–15 g) in the four *P* settings (30, 40, 50, and 60 W). The final AI value when a steam pop occurred under the same *P* of 30, 40, 50, and 60 W decreased for different baseline Rs (R to the pop, 30 W: *r* = 0.5431, *p* < 0.05; 40 W: *r* = 0.7509, *p* < 0.05; 50 W: *r* = 0.7326, *p* < 0.05; and 60 W: *r* = 0.8559, *p* < 0.05). The pattern in the level of the *R* drop and the steam pops was the opposite. The *R* drop during a steam pop under the multiple Ps of 30, 40, 50, and 60 W increased (30 W: *r* = 0.6318, *p* < 0.05; 40 W: *r* = 0.8811, *p* < 0.05; 50 W: *r* = 0.9107, *p* < 0.05; and 60 W: *r* = 0.9038, *p* < 0.05).

## Discussion

The principle behind RFCA is to convert a radiofrequency current into thermal energy and release it to the target myocardium at the same time, leading to tissue heating and cellular necrosis through the two types of energy: impedance heat and conductive heat. Although a study (11) found that there were no significant differences in lesion size if it reached the same target AI, with diverse *P* settings, CF, and the other factors. Our present study demonstrated that in RFCA, the surface diameter and the maximum diameter, depth, and volume of the lesions were strongly correlated with the AI. According to Joule's law, if the current, impedance, and duration remain unchanged, the heat generated by the ablation catheter remains unchanged, and the lesions are the same in theory. However, the effect of the human body is a variable resistance factor in the circuit; therefore, numerous studies have investigated the general baseline *R* in humans. These studies (12–14) have demonstrated that the value of *R* in humans fluctuates between 100 and 120 Ω. In the clinic, in certain areas with slow blood flow, the baseline *R* is higher than that in other areas (e.g., the coronary sinus or the pulmonary veins); therefore, the *R* in these veins can sometimes reach 180 Ω or even exceed 200 Ω. During ablation in the coronary sinus, there is a need to increase the irrigation fluid flow rate and reduce the power (30 W, 30 ml/min). However, sometimes it still cannot be ablated normally. It is necessary to increase the impedance limitation to release RF energy normally. Therefore, the characteristics of lesions formed under different baseline impedances are still worth studying.

Low power (20–40 W) with a relatively long duration (20–40 s) ablation is a common approach for ablating along the posterior wall of the left atrium in atrial fibrillation (AF) radiofrequency ablation (RFA) to reduce unintended thermal injury. Here, we observed the relationship between baseline impedance and lesion dimension using STSF with low *P* (30 W, AI = 350). The increase of baseline impedance was mainly reflected in the increase of maximum inner diameter, but the increase of ablation lesion depth was not obvious. Our data show that without steam pops, the increase of baseline impedance could make the lesion more uniform and extensive, and fewer ablation points can be achieved to obtain satisfactory linear or flake ablation of the lesion. The results of this *ex vivo* experiment suggest that the optimal baseline *R* should be between 100 and 140 Ω. This range of *R* load allows a considerable lesion size with no risk of tissue overheating and steam pop formation. These findings are consistent with the results of a previous report by Barkagan et al. (1). However, they changed the baseline *R* by adjusting the series *R* in the circuit instead of changing the *R* of normal saline in the container. Shapira-Daniels et al. changed the contact area between the negative plate and the skin (the parallel circuit reduces the impedance), so as to achieve the purpose of reducing the impedance (15). As a result, they found the opposite pattern that this study found: RFCA at a lower *R* resulted in increased tissue heating and led to larger lesion dimensions because of the increased current delivery (1). The author considered that due to the different conditions of the human body environment (such as electrolyte and blood cell concentration), the electrical conductivity and *R* in humans varied. In this experiment, the basic impedance was adjusted by adding purified water to 0.9% NaCl saline. This may be one of the reasons for the contrary outcome compared to previous experimental results because it reflects the difference of ablation focus size caused by the difference of baseline impedance in different individuals. The rise of impedance reflects the increase of local impedance in the gap between the catheter and the tissue, and the heat generated still increases to a certain extent. Therefore, the transmission of energy to the depth of the tissue is not obvious, and the maximum inner diameter increases.

Furthermore, it has been reported that the different types of perfusion fluid could also lead to different ablation lesion characteristics (16). Huang et al. (10) sought to characterize lesion formation characteristics using different irrigants. In their experiment,half normal saline ablation created larger lesions than normal saline and glucose water lesions were significantly larger than saline lesions, glucose yielded a deeper and narrower lesion, while saline yielded a wider ablation lesion because of current dispersion, and the partial current delivery to the surrounding tissue caused an efficient ablation lesion.

In addition, AI is useful to deliver adequate lesions but inadequate to forecast steam pops (8). Steam pops were associated with the boiling and popping at the tip and tissue avulsion and thrombus formation on the catheter tip (17). From a safety perspective, we found that when the power transmission reached 50 and 60 W in the HR group and the VHR group, the steam burst reaction of the STSF duct increased significantly. These findings suggest that high-power ablation is unsuitable for AI values >450 at high baseline impedance. In addition, when the *P* was 60 W and the *R* was 180–220 Ω, all points experienced a steam pop before AI = 500. We observed that the baseline impedance increases, may result in the local temperature of ablation rising too fast. This leads to the preferential conduction of RF toward tissue and subsequent higher current density inside the tissue, and some even fail to reach the target AI and, steam burst occurs. In this experiment, it can be seen that when a special AI value (*R* = 180–220 Ω, *P* = 40 W) occurs, the AI can reach 800 when a steam explosion occurs. The author speculates that this is because the adventitia of the ablation site is slightly thicker and there is local fatty tissue. The presence of adipose tissue may result in less RF energy transfer and lower caloric value, and thus a larger AI value can be achieved. For all steam pops, after incision, we could observe that the fracture direction of steam pops was usually parallel to the tissue surface. All steam pops made loud bursts, but in the HR and vHR groups, no obvious tissue fracture could be seen in the steam pops when in low power (30 W). It indicates that a steam pop at high power results in more serious damage to the tissue. In addition, small blood vessels can be seen under the skin in some steam pop cross-sections. Besides Nguyen et al. (17) used an *ex vivo* model consisting of viable bovine myocardium and found that a perpendicular catheter position yielded larger lesions, and the incidence of steam pops was observed more frequently in this position. Therefore, in clinical work, in order to increase the safety of ablation and minimize the vertical adhesion between the catheter and the tissue, the lateral adhesion method is used for ablation.

Fortunately, it is rare that the baseline impedance is > 200 Ω in clinic. If it occurs, the operator should avoid high-power and long-term ablation, and can choose to increase the perfusion fluid flow rate to reduce the local impedance. In particular, when the impedance increases, the corresponding target AI should not be set too high to avoid the occurrence of clinical adverse events. The authors of this study believe that it is essential to select the appropriate *P* setting and target AI in RFCA to master the characteristics of lesions.

## Limitations

The limitations of this experiment are as follows: first, in the clinic, RFCA may sometimes establish a respiratory gate to reduce the effect of respiratory movement. In this experiment, *ex vivo* tissue was used, and the catheter was manually fixed to ensure the catheter was close to the tissue during RFCA. Second, the experimental conditions only consider the *R* change, but these do not correspond to the relative electrical conductivity and osmotic pressure of the human body. Third, the physical properties of any preclinical animal differ from those of humans, and this ablation perspective should be evaluated clinically.

## Conclusion

Different baseline Rs led to different ablation lesion characteristics in RFCA, and the surface diameter, maximum diameter, depth, and volume of the lesions were strongly correlated with the AI. Larger lesions could be obtained under medium *P* and high baseline *R*. In addition, with increasing baseline *R*, the lesion size increased. The incidence of steam pops was strongly correlated with high baseline *R* and high P. The higher the baseline value of *R*, the greater the absolute value of the decrease in *R*. In conclusion, multiple factors can influence the characteristics of ablation lesions in RFCA. The ablation parameters must be individualized to patients to best meet their clinical needs.

## Data Availability Statement

The original contributions presented in the study are included in the article/supplementary material, further inquiries can be directed to the corresponding author/s.

## Ethics Statement

The animal study was reviewed and approved by Ethics Committee of First hospital of Shanxi Medical University.

## Author Contributions

LQ and MG: conception and design of the research and statistical analysis. LQ, MG, and XL: acquisition of data. LQ and NZ: analysis and interpretation of the data. RW, MG, and MS: obtaining financing. LQ: writing of the manuscript. MG and RW: critical revision of the manuscript for intellectual content. All authors read and approved the final draft.

## Funding

The National Natural Science Foundation of China (No. 82000426), the Natural Science Foundation of Shanxi Province (Nos. 201801D121222 and 201801D121337 to MG), and PhD Fund of the First Hospital of Shanxi Medical University (No. YB161702).

## Conflict of Interest

The authors declare that the research was conducted in the absence of any commercial or financial relationships that could be construed as a potential conflict of interest.

## Publisher's Note

All claims expressed in this article are solely those of the authors and do not necessarily represent those of their affiliated organizations, or those of the publisher, the editors and the reviewers. Any product that may be evaluated in this article, or claim that may be made by its manufacturer, is not guaranteed or endorsed by the publisher.
